# Biomarkers in the Light of the Etiopathology of IC/BPS

**DOI:** 10.3390/diagnostics11122231

**Published:** 2021-11-29

**Authors:** Jochen Neuhaus, Mandy Berndt-Paetz, Andreas Gonsior

**Affiliations:** 1 Department of Urology, Research Laboratory, University of Leipzig, 04103 Leipzig, Germany; mandy.berndt@medizin.uni-leipzig.de; 2 Department of Urology, University Hospital Leipzig AöR, 04103 Leipzig, Germany; andreas.gonsior@medizin.uni-leipzig.de

**Keywords:** interstitial cystitis/bladder pain syndrome (IC/BPS), urinary bladder immunity, gene expression, regulatory pathways, histology, miRNA

## Abstract

In this review, we focused on putatively interesting biomarkers of interstitial cystitis/bladder pain syndrome (IC/BPS) in relation to the etiopathology of this disease. Since its etiopathology is still under discussion, the development of novel biomarkers is critical for the correct classification of the patients in order to open personalized treatment options, on the one hand, and to separate true IC/BPS from the numerous confusable diseases with comparable symptom spectra on the other hand. There is growing evidence supporting the notion that the classical or Hunner-type IC (HIC) and the non-Hunner-type IC (NHIC) are different diseases with different etiopathologies and different pathophysiology at the full-blown state. While genetic alterations indicate close relationship to allergic and autoimmune diseases, at present, the genetic origin of IC/BPS could be identified. Disturbed angiogenesis and impairment of the microvessels could be linked to altered humoral signaling cascades leading to enhanced VEGF levels which in turn could enhance leucocyte and mast cell invasion. Recurrent or chronic urinary tract infection has been speculated to promote IC/BPS. New findings show that occult virus infections occurred in most IC/BPS patients and that the urinary microbiome was altered, supporting the hypothesis of infections as major players in IC/BPS. Environmental and nutritional factors may also influence IC/BPS, at least at a late state (e.g., cigarette smoking can enhance IC/BPS symptoms). The damage of the urothelial barrier could possibly be the result of many different causality chains and mark the final state of IC/BPS, the causes of this development having been introduced years ago. We conclude that the etiopathology of IC/BPS is complex, involving regulatory mechanisms at various levels. However, using novel molecular biologic techniques promise more sophisticated analysis of this pathophysiological network, resulting in a constantly improvement of our understanding of IC/BPS and related diseases.

## 1. Introduction

Interstitial cystitis/bladder pain syndrome (IC/BPS) is a disabling disease, with a reported prevalence of 52—500/100,000 in the female and 8—41/100,000 in the male population [1,2,3]. The full-blown disease has a significant social impact since participation in social activities is severely hampered. Up to date, no specific biomarkers have been found, allowing unequivocal diagnosis of the disease. Therefore, diagnosis largely relies on the exclusion of confusable diseases [4]. IC/BPS comprises the classic IC/BPS, presenting cystoscopically identifiable Hunner lesions of the urothelium (HIC), contrasting that the non-Hunner-type interstitial cystitis (NHIC) [5,6] and other molecular subtypes may emerge in the future. Furthermore, the etiopathology of IC/BPS is unknown. Since therapeutic options are limited, early detection of patients susceptible to IC/BPS is one of the most urgent clinical challenges. Here we summarize the current knowledge on potential biomarkers, linking them to potential etiopathology of IC/BPS.

## 2. Materials and Methods

We used PubMed (https://pubmed.ncbi.nlm.nih.gov/?db=PubMed (accessed on 22 November 2021)) and PubMed central searches (https://www.ncbi.nlm.nih.gov/pmc (accessed on 22 November 2021)) to identify articles related to bladder histology, immunology, cell- and molecular biology. We integrated these reports into a narrative review of biomarkers putatively of interest for further investigation of the etiopathology of IC/BPS and the definition of subgroups. The review does not claim to be complete, instead reflecting only the viewpoint of the authors.

## 3. Results

### 3.1. Genetic Factors—Potential Drivers of IC/BPS

Numerous studies suggest an indirect relationship of genetics and the special occurrence of IC/BPS. Frequent comorbidities such as allergies or autoimmune diseases imply a certain share of genetics on the development of IC/BPS (Table 1), and significant associations have been shown [7]. There is also significant evidence for cross-sensitization mechanisms which might drive one or the other organ disease [8]. However, up to date there is direct evidence of genetic alteration leading to IC/BPS.

Some studies suggest at least a genetic predisposition of IC/BPS. Monozygotic siblings developed significantly more frequently IC/BPS symptoms (five out of eight) than dizygotic siblings (none of 26 siblings). The risk of developing IC/BPS was 17 times higher in first-grade relatives [19]. However, a large study based on the Swedish Twin Registry including >25,000 twins revealed in women that the development of IC/BPS is substantially influenced by environmental factors, whereas genetic factors accounted for less than one-third of the observed variance [20].

On the other hand, Allen-Brady and coworkers reported a significant excess risk of near relatives for the IC/BPS associated conditions myalgia, fibromyalgia, and constipation. Most interestingly, there was also an excess risk for IC/BPS in patients with myalgia, fibromyalgia, and constipation, supporting the notion of a common underlying genetic factor of those diseases shared with IC/BPS [21]. In a recent genetic linkage analysis, the authors found IC/BPS-associated alterations in chromosome 3 and possibly several others on chromosomes 1, 4, 9, and 14, indicating a genetic predisposition for IC/BPS [22]. Larger studies are necessary for validation of these interesting data.

Single nucleotide polymorphisms (SNPs) analysis revealed a significant higher prevalence of the homozygote rs11127292 allele (genotype CC) in IC/BPS. Furthermore, the polymorphic allele rs6311 was detected in 90.5% of the patients with severe pain [23].

While validation of IC/BPS-associated genetic alterations is still pending, the results are promising for the development of novel diagnostic tests. Genetic predisposition might concern the immune system and relate to other autoimmune or allergic diseases.

### 3.2. Gene Expression, Networks and Signaling Pathways

Besides alterations in single genes, specific alterations in protein networks and signaling pathways have also been identified in IC/BPS patients by bioinformatic analysis of public expression data sets in the GEO database [24]. Despite the number of available expression data sets in IC/BPS was still very small (23 IC lesions vs. 9 normal tissues), the authors found 42 differentially expressed genes (DEGs). A total of 41 of those DEGs formed a protein-protein interaction network (PPI) of 41 knots, of which 11 genes were altered more than 10-times and could be addressed as central knot genes. Those were mainly cell-surface proteins and related to inflammation and immune system activation. In subgroup analyses, 12 DEGs were exclusively associated with HIC, while 27 DEGs were clearly associated with NHIC. Amongst others, the chloride voltage-gated channel 3 (CLCN3) was overexpressed [24]. This chloride channel is expressed in the smooth muscle and the urothelium and could trigger pain by spontaneous depolarization. Furthermore, overexpression of several genes of the protein S100 family were identified, playing a key role in NF-κB mediated inflammation. In addition, E2F1 and CCNA2, both associated with cell cycle, were upregulated in IC-lesions. However, their roles in IC/BPS remains unclear.

Gene expression is closely regulated by microRNAs (miRNAs) and disease associated alterations of miRNA expression have been detected, including urothelial carcinoma [25]. Recent studies have provided evidence for a significant role of miRNAs in inflammation and tumor. In IC/BPS, Arai et al. found upregulation of 163 and downregulation of 203 miRNAs in IC/BPS-patients compared to healthy controls. Especially members of the miR-320 family were affected, which regulate the expression of the transcription factors E2F1, E2F2 and TUB. Immunohistochemistry supported the overexpression of those transcription factors in IC/BPS [26].

Many miRNAs were upregulated in IC/BPS-patients, including miRNAs inhibiting the transcription of neurokinin receptor genes (TACR1 and TACR2), correlating with the downregulation of those neurokinin receptors at protein level [27]. Upregulation of miR-199a-5p in IC/BPS patients could impair the urothelial barrier via inhibition of the gene expression of several tight junction-associated proteins [28]. I In summary, miRNAs are promising molecules for the development of novel molecular diagnostic tests due to their broad involvement in cell proliferation, cell differentiation, inflammatory response, and fibrosis. Such biomarkers might significantly improve the currently tissue-based diagnosis of IC/BPS, suffering from very limited validity of pure histopathological evaluation (e.g., of the mast cell infiltration and fibrosis in bladder biopsies). Furthermore, the pleiotropic effects of miRNAs could also explain the numerous associations of IC/BPS with allergic diseases. Therefore, miRNAs may also prove a valuable new therapeutical approach.

As recently shown, long-noncoding RNA (i.e., maternally expressed gene 3 (MEG3)) is upregulated in HIC patients compared to a healthy control group and upregulates the endosomal toll-like receptor 7 (TLR7), a pattern recognition receptor detecting single strand RNAs (ssRNAs) from bacteria [29], viruses [30] and self-antigens [31], finally leading to the release of inflammatory cytokines in HIC bladders [32]. TLR7 was found to be upregulated in the urinary bladders of HIC patients [33]. Interestingly, the action of MEG3 is enhanced by the downregulation of miR-19a-3p, competing on the RNA binding site in MEG3 [32]. These findings suggest that HIC etiopathology involves bacterial or viral infection, requiring participation of B-cells, macrophages, and dendritic cells [34].

### 3.3. Occult Urinary Tract Infections and the Microbiome

Several recent studies support the view that IC/BPS might be triggered by occult uropathogens, bacteria or viruses. As shown by Aydogan et al., a special microbiologic culture method and real-time polymerase chain reaction (RT-PCR) can detect different uropathogenic bacteria in the urine of symptomatic IC/BPS patients, previously diagnosed with sterile urine sample [35]. Especially the cell wall-deficient, so-called L-form bacteria were detected, which are susceptible to different osmolarities in culture media. This study supports previous findings that only 33% of the bacterial population were detectable by standard culture methods [36].

An altered microbiome could promote the establishment of pathogenic microbes. However, a comprehensive analysis of the microbiome in urine samples and vaginal smear of 41 pre-menopausal women diagnosed with IC/BPS did not reveal any differences compared to unaffected controls [37]. Of interest, however, was a higher correlation between urine and vaginal samples, indicating a reduced diversity of the microbiome. This could be interpreted due to the impaired barrier function in the epithelium. In addition, age-related alterations of the urinary microbiome, and especially the changes in Lactobacillus may contribute to recurrent urinary tract infections in post-menopausal women. The role of the microbiome in IC/BPS is still unclear [38]. However, since those studies relied on standard cultivation methods, novel extended cultivation could lead to new insights.

### 3.4. Virus Infections in IC/BPS?

New evidence has accumulated indicating a role of viral infections in IC/BPS. As shown by Jhang et al., the Epstein-Barr virus (EBV) was present in 50% of the patients with Hunner-lesion (HIC), but in only 8.6% of the NHIC patients. In controls, the authors could not detect EBV-RNA [39]. Also, BK polyoma viruses (BKPyV), which can induce hemorrhagic cystitis [40], were found in the urine of IC/BPS patients and could play a role in the etiopathology of IC/BPS [41]. Occult bacterial infection, persisting in macrophage was already demonstrated in endocarditis [42]. If this could be a blueprint for the occult persisting viral infection in IC/BPS still has to be investigated.

Recently, evidence was found for a virus-induced cystitis in COVID-19 patients. The infection of a subpopulation of urothelial cells expressing the angiotensin-converting enzyme 2 (ACE2) receptor could indeed be responsible for the occurrence of a de novo urgency in patients showing IC/BPS typic sterile urine cultures. However, it is unclear whether the infection happens luminal via the urine or basal viremic. A local endotheliitis could also play a role and would also provoke local hypoxia in the tissue [43]. In addition, the urine of COVID-19 patients with sterile de novo cystitis showed elevated cytokine levels, which could be produced by the urothelial cells or come from renal excretion [44]. Elevated levels of cytokines and chemokines were already described in patients with idiopathic urgency and inflammatory bladder alterations [45].

### 3.5. Mast Cells and Lymphocytes as Biomarkers

Mast cell infiltration correlates with the infiltration of other immune cells and cannot serve as a good differential diagnostic criterion [46]. Neither in the lamina propria nor in the detrusor significant differences were evident in a recent systematic immunohistochemical study [47]. However, the focal clonal expansion of B-lymphocytes and the occurrence of plasma cells seem to be characteristic for HIC [6], raising the question what may be the relevant immunologic processes evoked by the clonal B-cell response in IC/BPS. Clonal B-cell expansion has been observed in several autoimmune diseases, such as Sjögren’s syndrome [48] and rheumatoid arthritis [49], but also may be triggered by bacterial infection with Helicobacter pylori [50] or by the Epstein-Barr virus [51]. In a recent study, almost 60% of the IC/BPS patients turned out to have an occult Epstein-Barr virus infection [39]. This might open a new causality chain of the development of IC/BPS, especially of HIC. Interestingly, the virus was mainly located in T-cells, suggesting that not only B-cells but also T-cells are involved [52].

### 3.6. Platelet Activating Factor (PAF)

PAF is the trivial name of a phospholipid, inducing thrombocyte aggregation, being a highly potent pro-inflammatory mediator produced and released by many cells, including epithelial cells and leucocytes. In patients with anaphylaxis PAF is upregulated and at the same time the deactivating key enzyme PAF-acetylhydroxylase being downregulated [53]. PAF has been related to several other allergic diseases, since it triggers immunologic reactions, amongst others the production of reactive oxidative species (ROS), the rising of inducible nitric oxide synthase (iNOS), cyclooxygenase-2 (COX-2), and the expression of the pro-inflammatory cytokines IL-6 and TNF. Chronic stimulation can modulate the PAF production. Cigarette smoke can induce the production of PAF and of its receptor PAFR in bladder urothelium. PAF was also present in the urine of IC/BPS patients and increased with cigarette smoking [54]. Furthermore, PAF can enhance the production of MMPs [55,56], which are able to reduce the expression of tight junction proteins, finally resulting in dysfunction and disruption of the epithelial barrier [54,57,58]. While the causality of elevated PAF levels and urothelial barrier damage has been well established, studies investigating the possible use of PAF as an early biomarker of IC/BPS are still pending.

### 3.7. The Role and Regulation of Vascular Endothelial Growth Factor (VEGF)

Stress can modulate bladder function and its role in triggering IC/BPS symptoms is frequently reported. At molecular level, the corticotropin releasing hormone receptor (CRHR) has been identified as a sensor of stress. Two CRH-receptors, located at different gene loci are known. IC/BPS patients showed a significant higher expression of CRHR1 but lower expression of CRHR2 in the urothelium and the lamina propria [59]. The CRHR1 expression positively correlated with the expression of the nerve growth factor (NGF) but negatively with E-cadherin. The authors also reported that the O’Leary-Sant-Score and the clinical symptom scores, including the ICSI, ICPI and VAS were correlated to the CRHR expression [59]. While the mechanism of CRHR signaling in urothelial cells is still unclear, the changes in the receptor expression suggest that CRH-related peptides could play a role in the IC/BPS etiopathology.

Interestingly, CRH can stimulate mast cells to release VEGF [60]. Thereby, infiltrating mast cells are a source of VEGF. VEGF regulates the angiogenesis and lymphangiogenesis, and diminished VEGF activity can lead to vascular abnormalities which may be regredient onto substitution of VEGF. In vitro, VEGF stimulates the proliferation and migration of endothelial cells and enhances the vascular permeability. It has been shown that VEGF also has neuroprotective activity. In consequence, acute stress can increase the vascular permeability and may alter nerve fibers in the urinary bladder [61,62].

Significant higher levels of VEGF were detected in biopsies of Hunner-type lesions and in tissue samples of petechial bleedings compared to samples without petechiae and in healthy controls. Very high VEGF levels cause immature microvessel formation with inadequate pericyte coverage, enhancing the risk or hemorrhage [61].

Thus, alterations of the CRH-VEGF-axis might be a useful parameter of IC/BPS, but larger studies are required for validation.

### 3.8. Alterations of the Urothelial Barrier—A Hallmark of IC/BPS

The healthy urothelium consists of several layers of urothelial cells, covered by flat, hexagonal umbrella cells, and armed with a glycosaminoglycan (GAG) layer. This GAG layer is part of the urothelial barrier, preventing urine, bacteria, cations, and other urine components to infiltrate into the deeper urinary bladder wall. Damage of this urothelial barrier is a hallmark of IC/BPS in human and in animals (feline interstitial cystitis, FIC) [63,64].

Molecular alterations of the urothelial barrier have been described in the dysregulated expression of tight junction proteins, regulating the paracellular transport, and disruption of tight junctions can directly be related to pain sensation in the bladder. The regulation and molecular pathology of the system is complex as involving several levels of regulation. Sanchez Freire et al. found in IC/BPS altered expression levels of micro-RNAs (miRNAs) targeting genes involved in signal transduction, muscle contraction, and epithelial permeability [65]. The authors found downregulated mRNA levels of Zonula occludens-1 (ZO-1), Junctional adhesion molecule 1 (JAM-1), Occludin, and Claudin 1, indicating an impaired tight junctional barrier in IC/BPS bladders. Furthermore, the mRNA expression of Claudin 4, responsible for the formation of “tight” tight junctions [66], was unaltered, while the expression of Claudin 2, which makes “leaky” tight junctions by forming paracellular channel was significantly overexpressed [65]. Most interesting, Claudin 2 forms cation-selective paracellular pores [67], allowing the passage of sodium and potassium ions. Together with the upregulation of the Acid sensing ion channels (ASIC2a and ASIC3) in IC/BPS patients [27] the overexpression of Claudin 2 could directly trigger bladder pain.

The tachykinin receptors (NK1R and NK2R) were downregulated in IC/BPS, but Bradykinin (B1) and Cannabinoid receptors, and the transitional receptor potential receptor M8 (TRPM8) [68] were upregulated as it was shown for several muscarinic receptors (M3-M5) at mRNA and protein level [27,69].

While the differences in expression levels are not easily explainable, the study of Sanchez Freire et al. provides some insights into the complex regulatory network. The authors could show that chronic stimulation of the NK1R by substance P resulted in downregulation of the receptor and that this was counteracted by upregulation of certain regulatory miRNAs (miR-449b and miR-500) in cell culture. In human they confirmed the correlation of miRNA-regulation and downregulation of NK1R mRNA in IC/BPS patients. By database research they identified several of the proteins altered in IC/BPS as targets of those miRNAs [65].

## 4. Conclusions

The etiopathology of IC/BPS is still unclear. Evidence accumulated from using modern state of the art approached to molecular biology and cell biology. Especially, our growing understanding of the complex gene regulation mechanisms at the level of miRNA and cellular crosstalk with immune system has greatly improved our view in IC/BPS.

In summary, miRNA seem to be the top level in the regulatory network responsible for the changes in protein expression in IC/BPS. Specific test panels should be evaluated for their performance in the detection of early onset of IC/BPS and may also prove valuable for differential diagnosis of IC/BPS.

A single decisive cause becomes more and more improbable, and several subtypes of IC/BPS crystallizes, furthermost HIC and NHIC. The latter will probably split into several subgroups defined by their specific pathological causality. New biomarkers are at the horizon shedding light into the confusing situation of IC/BPS etiopathology (Table 2).

## Figures and Tables

**Table 1:** Diseases overrepresented and frequently associated with IC/BPS.

Comorbidity	References
Allergies and autoimmune disorders	[9]
Asthma (especially the non-allergic type)	[10]
Sjögrens’s syndrome	[11,12,13]
Atopic dermatitis	[9]
Lupus erythematosus	[14]
Fibromyalgia	[9,13,15]
Rheumatoid arthritis	[9,15]
Chronic fatigue	[13]
Endometriosis	[16]
Irritable bowel syndrome, Colitis ulcerosa	[9,17]
Hashimoto’s thyroiditis and Hyperthyroidism	[9,15,18]
Psoriasis	[9]

**Table 2:** Potential biomarkers related to etiopathology of IC/BPS.

Focus	Marker	Gene/Protein	Effect	References
Predisposition	Chromosome			[22]
	3p13-p12.3	*CNTN3*	sensory processing/pain	
	1p21-q25	*NGF*	nerve proliferation	
		*IL6*	inflammation	
		*CRP*	inflammation	
	3p21.1-p14.3	*CACNA2D3	neural activity (brain)	
	4q12-q13	*PDGFRA	proliferation, development	
	9p24-p22	*IL33*	innate immunity, mast cell activation/proliferation	
	14q24-q31	*FOS*	inflammation	
	SNP			
	rs11127292	*MYT1L*	neuronal differentiation	[23]
	rs6311	*HTR2A*	pain	
	rs1799971	*OPRM1*	pain	
Early detection	PAF and PAFR	iNOS, COX-1, IL-6, TNF	urothelial barrier and vascular integrity	[54]
	CRH-VEGF-axis CRHR		microvessel formation	[61]
Differential	*CLCN3*	CLCN3	pain	[24]
diagnosis	*S-100 gene family*	S-100 proteins	inflammation	
(HIC vs. NHIC)	*E2F1*	E2F1	cell cycle	
	lncRNA MEG3	TLR7	immunity	[32]
	miR-19a-3p	MEG3	immunity	
	B-lymphocytes, plasma cells		clonal expansion infiltration	[6]
	CRHR		vascular integrity, microvessel formation	[59]
Diagnosis/Prognosis	miR-320 family	*E2F1/2, TUB* *TACR1/2*	cell cycle pain	[26]
	miR-199a-5p	PALS1	tight junction formation, urothelial cell polarity	[27,28]

Abbreviations (alphabetical): AP-1, Transcription factor subunit; CACNA2D3, Voltage-dependent calcium channel subunit alpha-2/delta-3; CCNA2, Cylin-A2; CLCN3, Chloride voltage-gated channel 3; E2F1/2, Transcription factor E2F1/2; CNTN3, Contactin-3; CRH, Corticotropin releasing hormone; CRP, C-reactive protein; FOS, Fos Proto-Oncogene, TR2A, 5-Hydroxytryptamine Receptor 2A; IL33, Interleukin 33; IL6, Interleukin 6; lncRNA, long non-coding RNA; MEG3, Maternally expressed gene 3; MYT1L, Myelin transcription factor 1-like protein; NGF, Nerve growth factor; OPRM1, Opioid receptor Mu 1; PAF, Platelet-activating factor; PAFR, PAF receptor; PALS1, Protein PALS1; PDGFRA, Platelet-derived growth factor receptor alpha; SNP, single nucleotide polymorphism analysis; TACR1/2, Substance-P receptor; TUB, Tubby protein homolog; VEGF, vascular endothelial growth factor; * added by author.
